# COnTORT: COmprehensive Transcriptomic ORganizational Tool for Simultaneously Retrieving and Organizing Numerous Gene Expression Data Sets from the NCBI Gene Expression Omnibus Database

**DOI:** 10.1128/MRA.00587-20

**Published:** 2020-06-18

**Authors:** Kevin S. Myers, Michael Place, Daniel R. Noguera, Timothy J. Donohue

**Affiliations:** a Wisconsin Energy Institute and Great Lakes Bioenergy Research Center, University of Wisconsin—Madison, Madison, Wisconsin, USA; b Department of Civil & Environmental Engineering, University of Wisconsin—Madison, Madison, Wisconsin, USA; c Department of Bacteriology, University of Wisconsin—Madison, Madison, Wisconsin, USA; Loyola University Chicago

## Abstract

We introduce COnTORT (**CO**mprehensive **T**ranscriptomic **OR**ganizational **T**ool), a publicly available program that retrieves all available gene expression data and associated metadata for an organism from the National Center for Biotechnology Information (NCBI) Gene Expression Omnibus (GEO) database. The data are compiled into text files that can be used for downstream bioinformatic applications.

## ANNOUNCEMENT

**FIG 1:**
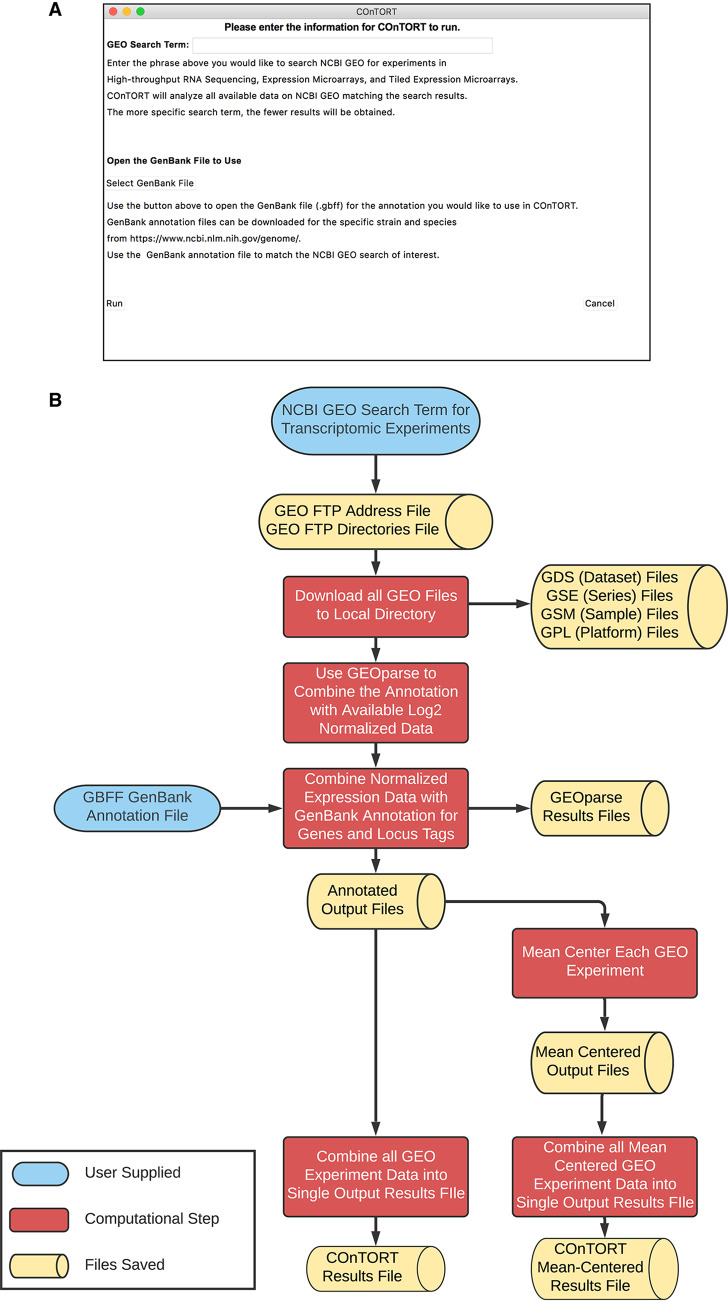
Overview of the COnTORT process. (A) COnTORT interface where the user enters the query to search NCBI GEO and selects the GenBank file to be used. (B) Flowchart of the COnTORT program. NCBI GEO search results are parsed, and all GEO data are downloaded. Normalized gene expression data from the NCBI GEO are retrieved. The normalized files are organized with the annotation from the GenBank file, mean-centered, and combined into COnTORT result files.

The National Center for Biotechnology Information (NCBI) Gene Expression Omnibus (GEO) database is a centralized repository for millions of functional genomic data sets, including gene expression data from both microarrays and high-throughput sequencing (1–3). While there are tools from both the NCBI and others (e.g., GEOquery [4] and GEOparse [https://geoparse.readthedocs.io]) to allow for access to individual data sets, there is no publicly available tool that will allow users to bulk-retrieve GEO data and organize all the available gene expression data for a given organism. Overcoming this limitation will increase the use of NCBI GEO data for large-scale analyses using the available gene expression data. Here, we introduce COnTORT (**CO**mprehensive **T**ranscriptomic **OR**ganizational **T**ool), a publicly available Python3 program that facilitates the retrieval of all publicly available gene expression data derived from a search in the NCBI GEO. COnTORT requires only a GenBank gene annotation file for the organism of choice (5) and will perform a search of the NCBI GEO entered by the user upon running the program (Fig. 1). This query can be as specific or general as desired, meaning the results can be narrow or broad to answer a particular question.

COnTORT downloads all NCBI GEO data associated with the search term provided and uses these data to produce tab-delimited text files containing the organized results of the gene expression data. The first five columns contain the annotation information from the GenBank file (locus tag, old locus tag, gene name, gene synonyms, and product), and the remaining columns contain the gene expression data for each experimental sample. One output file will mean-center the data based on experiments to control for differences between experiments, while the second output file will not mean-center the data. These output files are in a format that can be used for virtually any downstream bioinformatic application.

Acquiring numerous bacterial samples takes about 20 min, while retrieving data from yeast or humans, with much larger genomes, takes around an hour to complete on a standard desktop computer. COnTORT thus provides a significant savings in time and effort for researchers in gaining access to large numbers of gene expression data sets from a single organism. We have used COnTORT files to correlate expression of genes across a large number of diverse data sets to identify new targets of transcription factors in bacteria. While COnTORT builds upon critically important tools such as GEOparse and GEOquery, and indeed uses GEOparse, it allows researchers to harness the power of the millions of gene expression data sets in the NCBI GEO database.

COnTORT is written for the analysis of gene expression data. However, because the scripts are publicly available, changes can be made to allow COnTORT to download and combine all the available data from chromatin immunoprecipitation (ChIP)-chip or ChIP-seq experiments. Further, COnTORT can be modified to acquire data in any online database, such as those housed by the European Bioinformatics Institute (6). While COnTORT is a valuable tool for the analysis of gene expression data, it also provides a platform on which additional tools and analyses can be built.

### Data availability.

COnTORT is available at https://github.com/GLBRC/contort and https://pypi.org/project/contort. A detailed description and tutorial are available at https://github.com/GLBRC/contort/blob/master/Additional_Information.md.
